# Neurochemical and Behavioral Profiling in Male and Female Rats of the Psychedelic Agent 25I-NBOMe

**DOI:** 10.3389/fphar.2019.01406

**Published:** 2019-12-12

**Authors:** Cristina Miliano, Matteo Marti, Nicholas Pintori, Maria Paola Castelli, Micaela Tirri, Raffaella Arfè, Maria Antonietta De Luca

**Affiliations:** ^1^Department of Biomedical Sciences, University of Cagliari, Cagliari, Italy; ^2^National Institute of Neuroscience (INN), Universirty of Cagliari, Cagliari, Italy; ^3^Department of Morphology, Experimental Medicine and Surgery, Section of Legal Medicine and LTTA Centre, University of Ferrara, Ferrara, Italy; ^4^Collaborative Center for the Italian National Early Warning System, Department of Anti-Drug Policies, Presidency of the Council of Ministers, Rome, Italy; ^5^Institute of Public Health, Section of Legal Medicine, Università Cattolica del Sacro Cuore, Rome, Italy

**Keywords:** novel psychoactive substances, sex differences, dopamine, serotonin, behavior

## Abstract

4-Iodo-2,5-dimethoxy-*N*-(2-methoxybenzyl)phenethylamine (25I-NBOMe), commonly called “N-Bomb,” is a synthetic phenethylamine with psychedelic and entactogenic effects; it was available on the Internet both as a legal alternative to lysergic acid diethylamide (LSD) and as a surrogate of 3,4-methylenedioxy-methamphetamine (MDMA), but now it has been scheduled among controlled substances. 25I-NBOMe acts as full agonist on serotonergic 5-HT2A receptors. Users are often unaware of ingesting fake LSD, and several cases of intoxication and fatalities have been reported. In humans, overdoses of “N-Bomb” can cause tachycardia, hypertension, seizures, and agitation. Preclinical studies have not yet widely investigated the rewarding properties and behavioral effects of this compound in both sexes. Therefore, by *in vivo* microdialysis, we evaluated the effects of 25I-NBOMe on dopaminergic (DA) and serotonergic (5-HT) transmissions in the nucleus accumbens (NAc) shell and core, and the medial prefrontal cortex (mPFC) of male and female rats. Moreover, we investigated the effect of 25I-NBOMe on sensorimotor modifications as well as body temperature, nociception, and startle/prepulse inhibition (PPI). We showed that administration of 25I-NBOMe affects DA transmission in the NAc shell in both sexes, although showing different patterns; moreover, this compound causes impaired visual responses in both sexes, whereas core temperature is heavily affected in females, and the highest dose tested exerts an analgesic effect prominent in male rats. Indeed, this drug is able to impair the startle amplitude with the same extent in both sexes and inhibits the PPI in male and female rats. Our study fills the gap of knowledge on the behavioral effects of 25I-NBOMe and the risks associated with its ingestion; it focuses the attention on sex differences that might be useful to understand the trend of consumption as well as to recognize and treat intoxication and overdose symptoms.

## Introduction

The appearance of new psychoactive substances (NPS) is changing the trend in drug use worldwide (Orsolini et al., 2015; Miliano et al., 2016; UNODC, 2016; Corkery et al., 2017; Orsolini et al., 2017), which includes also their safely perceived purchase on the Internet (Miliano et al., 2018). As a matter of fact, in the last decades the classical drugs of abuse are being progressively substituted by their “legal synthetic alternatives” or simply co-abused with these powerful substances. In 2014, phenethylamines were the second most used class of NPS, after synthetic cannabinoids (UNODC, 2014; UNODC, 2016), while now the trend is moving to synthetic opioids (EMCDDA, 2019). These compounds are usually abused for their psychedelic and entactogenic effects mainly by people who attend electronic dance music, parties at nightclubs, and festivals (Palamar et al., 2016; Schifano et al., 2016; Palamar et al., 2017) in order to reach a “dissociative state” from reality, which unfortunately might lead to severe clinical issues (Schifano et al., 2019). Phenethylamines are a large family of compounds that are molecular variants of the core compounds, i.e., amphetamines, 3,4-methylenedioxy-methamphetamine (MDMA), etc. (Le Roux et al., 2015). The *N*-benzylmethoxy derivatives of the 2C hallucinogens (i.e., 2C-I, 2C-B, and 2C-C), commonly called NBOMes (4-Iodo-2,5-dimethoxy-*N*-(2-methoxybenzyl)phenethylamine), are probably the most famous; they used to be marketed as a legal lysergic acid diethylamide (LSD), with aliases such as “Smiles” and “N-bombs.” They act as full agonist on human and rat 5-HT2A receptors (Ki = 0.044 nM and Ki = 0.087 nM, respectively), with high affinity (Braden et al., 2006). As a consequence, low doses of the order of 50 µg are able to produce psychoactive effects (Kyriakou et al., 2015; Suzuki et al., 2015). They are usually ingested sublingually, orally, by insufflations, rarely intravenously, and it seems to be active at doses as low as 50–250 µg, but the typical dose range is 500–800 µg (Halberstadt and Geyer, 2014). The duration of action depends on the route of administration, ranging from 4–6 h (insufflation) to 6–10 h (sublingual). According to analysis performed in seizures of these products, 25I-NBOMe seems to be the most present compound. The central effects of these substances are due to the activation of 5-HT2A receptors, which are heavily expressed in cortical and forebrain areas, various brainstem nuclei, and the hippocampus (Cornea-Hébert et al., 2002), but side effects are both central and peripheral. Indeed, overdoses of “N-Bomb” can cause several toxicological effects such as tachycardia, hypertension, seizures, and agitation persisting for up to 3 days (Hill et al., 2013; Kelly et al., 2013; Rose et al., 2013; Stellpflug et al., 2014; Hieger et al., 2015). Several intoxication cases and some fatalities have been reported after the recreational use of 25-NBOMes (Walterscheid et al., 2014; Andreasen et al., 2015; Laskowski et al., 2015; Kueppers and Cooke, 2015; Shanks et al., 2015; Suzuki et al., 2015; Adamowicz et al., 2016; Morini et al., 2017). Therefore, all the 2C *N*-benzyl-methoxy derivatives were banned and scheduled as illegal drugs in many countries, such as Italy, USA, Canada, Russia, Sweden, and China (https://www.erowid.org/chemicals/2ci_nbome/2ci_nbome_law.shtml). Furthermore, the recurring use of these potent 5-HT2A agonists may contribute to develop seizures and the serotonin syndrome (Bosak et al., 2013), an excessive serotonergic activation that results in specific clinical signs, such as tremor, diarrhea, and delirium, neuromuscular rigidity, and hyperthermia in life-threatening cases (Boyer and Shannon, 2005). Despite the widespread use of these compounds, and the effects reported in humans, there is a lack of knowledge about their behavioral or toxicological effects. The serotonergic psychedelic effects of this compound have been confirmed by behavioral responses such as head twitch in C57BL/6J mice (NBOMe 0.1–1 mg/kg, s.c.) (Halberstadt and Geyer, 2014), wet dog shakes, and back muscle contraction (0.01–3 mg/kg, s.c.) in rats (Elmore et al., 2018), and all these effects were prevented by the administration of the selective 5-HT2A antagonist M100907 (Halberstadt and Geyer, 2014; Elmore et al., 2018). Indeed, 25I-NBOMe time-dependently and dose-dependently decreased locomotor activity in mice (Eshleman et al., 2014; Gatch et al., 2017) and showed full substitution of LSD in rats drug discrimination and more than 50% of appropriate responding in MDMA-trained rats (Eshleman et al., 2014). The MDMA-like action of 25I-NBOMe has been also confirmed *in vitro* since it acts inhibiting the monoamine reuptake transporters with different IC_50_ (hSERT, IC_50_ = 4.3 µM; hDAT, IC_50_ = 75 µM; hNET, IC_50_ = 19 µM) in HEK 293 cells (Zwartsen et al., 2017). Recently, it has been demonstrated that this compound increases dopamine (DA) and serotonin (5-HT) in the frontal cortex of male Wistar–Han rats when administered at 3 mg/kg subcutaneously (Herian et al., 2019). Moreover, DA levels in mice synaptosomal striatal fractions were increased by *in vitro* administration of this substance, and male mice showed an increased conditioned place preference when injected with 25I-NBOMe (0.3 mg/kg, i.p.) (Jeon et al., 2019). Conversely, male Sprague–Dawley rats did not press the active lever to obtain an infusion of 25I-NBOMe (Jeon et al., 2019). Collectively, the results available so far suggest a putative abuse liability of this compound, but further investigations on the neurochemical and behavioral effects seem to be necessary. Therefore, the aim of this study was to evaluate the effect of 25I-NBOMe on DA and 5-HT transmissions, performing *in vivo* microdialysis in three terminal areas strongly involved in reward and drug seeking [nucleus accumbens (NAc) shell and core and medial prefrontal cortex (mPFC)]. Moreover, several behavioral tests were performed to assess sensorimotor impairment as well as the risk of developing hyperthermia and have an altered nociceptive response under the effect of 25I-NBOMe. Additionally, due to the possibility to develop drug use-related psychotic disorders, we included an analysis of the acoustic startle reflex (prepulse inhibition, PPI) that is considered a marker of vulnerability for neuropsychiatric disorders (Siegel et al., 2013; Marti et al., 2019). Moreover, sex differences in drug addiction behavior have been extensively reported in both humans and rodents (Becker et al., 1982; Jackson et al., 2006; Zhao and Becker, 2010; Cummings et al., 2014; Fattore et al., 2014). The reason of such great differences is due to dimorphisms in the anatomy of the reward brain circuits (Walker et al., 2012), differences in the intrinsic properties of DA neurons (Melis et al., 2013), as well as ovarian hormone fluctuations (Becker and Hu, 2008; Castelli et al., 2014).Considering the lack of data about the effects of this compound on females, we decided to perform the entire experimental study in both male and female rats in order to underline possible sex differences. In addition, in order to try to explain why adolescent girls seem to be more susceptible at intense negative psychoactive effects of MDMA (Liechti et al., 2001), and generally more vulnerable to develop hallucinogen dependence (Wu et al., 2009; Wu et al., 2010), in an initial stage of the study, we investigate the relationship among different estrous cycle phases and extracellular DA and 5-HT levels in response to acute 25I-NBOMe administration.

## Materials and Methods

### Animals

Male and female Sprague–Dawley rats, weighing 275–300 g (Harlan Italy), were used for *in vivo* microdialysis and behavioral tests. Rats were housed four per cage, in standard plastic cages with wood chip bedding, at temperature of 22 ± 2°C and 60% humidity and under a 12-h light/dark cycle (lights on from 7.00 a.m.). Tap water and standard laboratory rodent chow (Mucedola, Settimo Milanese, Italy) were provided *ad libitum* in the home cage. All animal experiments were carried out in accordance with the Guidelines for the Care and Use of Mammals in Neuroscience and Behavioral Research according to Italian (D.L. 116/92 and 152/06) and European Council directives (609/86 and 63/2010) and in compliance with the approved animal policies by the Ethical Committee for Animal Experiments (CESA, University of Cagliari) and the Italian Ministry of Health (Aut. N. 162/2016- PR; Aut. N.352/2015-PR). All animals were handled once daily for 5 min for five consecutive days before the beginning of the behavioral tests. We made all efforts to minimize pain and suffering and to reduce the number of animals used.

### Substances and Doses

25I-NBOMe was purchased from LGC Standards S.r.l. (Milan, Italy), dissolved in 2% EtOH, 2% Tween 80, and 96% saline, and administered intraperitoneally (3 ml/kg) at different doses. A wider range of doses of 25I-NBOMe (0.1, 0.3, 0.5, and 1.0 mg/kg, i.p.) were chosen for behavioral tests in order to assess which was the lower effective dose both in male and female rats. The most effective doses (0.3 and 1.0 mg/kg, i.p.) were chosen for microdialysis experiments in order to minimize the number of rats.

### Determination of the Estrous Cycle Phases in Female Rats

Before starting experiments, the estrous cycle of female rats was monitored every day for 15 days, collecting vaginal smears in the early morning (between 8:00 and 9:00 a.m.). Vaginal secretion was collected by flushing into the vagina and out (two to three times) 20 µl of saline (NaCl 0.9%). The pipette tip was inserted gently and not deeply in order to avoid cervical stimulation (Cora et al., 2015). One vaginal fluid drop per rat was placed on glass slides. Unstained material was observed by bright-field microscopy, with ×20 and ×40 objective lenses (Marcondes et al., 2002). Observing the cytology of vaginal smear, it is possible to discriminate three cell types, and it is well established that the proportion among them corresponds to a particular phase of estrous cycle in rodents (Goldman et al., 2007; Cora et al., 2015). In this way, we were able to determine the following: proestrus (predominant nucleated epithelial cells); estrus (anucleuated cornified cells); metestrus (leukocytes, cornified, and nucleated epithelial cells in the same proportion); and diestrus (predominant little round leukocytes) (data not shown).

### *In Vivo* Microdialysis Studies

#### Surgery

Male and female Sprague–Dawley rats (275–300 g; Harlan, Italy) were anaesthetized with isoflurane gas and maintained under anesthesia using a breathing tube under a scavenging system while placed in a stereotaxic apparatus and implanted with vertical dialysis probes prepared as previously described (De Luca et al., 2015) with 1.5 or 3 mm dialyzing portion for NAc or mPFC, respectively. According to the rat brain atlas of Paxinos and Watson (1998), animals were implanted in the NAc shell (A +2.2, L +1.0 from bregma; V −7.8 from dura) or core (A +1.4, L +1.6 from bregma; V−7.6 from dura), or in the mPFC (A +3.7, L +0.8 from bregma; V −5.0 from dura).

#### Analytical Procedure

On the day following surgery, animals were connected to an infusion pump and probes were perfused with Ringer’s solution (147 mM NaCl, 4 mM KCl, and 2.2 mM CaCl_2_) at a constant rate of 1 µl/min. After a washout of 1 h, dialysate samples (20 µl) were collected every 20 min and injected into an HPLC equipped with a reversed-phase column (C8 3.5 um, Waters, USA) and a colorimetric detector (ESA, Coulochem II; ESA-CDS software) to quantify DA and 5-HT. The electrodes of the analytical cell were set at +125 mV (oxidation) and −175 mV (reduction) to detect dopamine and at −175 mV (oxidation) and +220 mV (reduction) to detect serotonin. The mobile phase contained 50 mM NaH_2_PO_4_, 0.1 mM Na_2_EDTA, 0.5 mM n-octyl sulfate, and 15% (*v*/*v*) methanol to evaluate dopamine concentration and 22% (*v*/*v*) methanol for serotonin detection (the pH of mobile phase was adjusted with Na_2_HPO_4_ to 5.5 and 5.7 for dopamine and serotonin, respectively). The sensitivity of the assay for DA/5-HT was 5 fmol/sample. Basal dialysate were collected until DA and 5-HT levels did not differ more than 10% in three consecutive samples. The average value was considered as the basal levels of DA/5-HT. The animals were treated with saline or 25I-NBOMe and monoamine levels were monitored for 2 h after the treatment. At the end of the experiment, animals were sacrificed and their brains removed and stored in formalin (8%) for histological examination to verify the correct placement of the microdialysis probe.

#### Statistical Analysis of Microdialysis Experiments

All the numerical data are given as mean ± SEM. Data were analyzed by utilizing one-way ANOVA or repeated measures ANOVA (two-way and three-way). Results from treatments showing significant overall changes were subjected to Tukey’s tests for *post hoc* comparisons, with significance at *p* < 0.05.

### Behavioral Studies

The differential effects of 25I-NBOMe were investigated using a battery of behavioral tests widely used in studies of “safety-pharmacology” for the preclinical characterization of NPS in rodents (De Luca et al., 2015; Vigolo et al., 2015; Ossato et al., 2015; Ossato et al., 2016; Canazza et al., 2016; Canazza et al., 2017; Giannotti et al., 2017; Fantinati et al., 2017; Marti et al., 2019). To reduce the animal’s stress induced by manipulation, and to confirm the stability and reproducibility over time of the responses of our tests, animals were trained two times per week for 2 weeks before the pharmacological treatment. All experiments were performed between 8:30 a.m. and 2:00 p.m. Experiments were conducted in blind by trained observers working together in pairs (Ossato et al., 2016). The behavior of rats (sensorimotor responses) was videotaped and analyzed off-line by a different trained operator that gives test scores. The behavioral tests were performed in a consecutive manner using six rats per treatment, according to the following sequence: measures of visual object responses (frontal and lateral views), overall tactile response (pinna, vibrissae, and corneal reflexes), acoustic response, measures of core body temperature (rectal measurement), visual placing response, and determination of the mechanical (tail pinch) acute pain. Despite the repetition of tests during the time, no changes in parameters such as body core temperature and responses to noxious stimuli, which are sensitive to stressful situations (Adriaan Bouwknecht et al., 2007; Kozlov et al., 2015), have been observed in naive animals and in saline/vehicle-treated animals. The startle and prepulse inhibition studies, instead, were performed on another cohort of rats using seven animals per treatment.

#### Sensorimotor Studies

We studied the voluntary and involuntary sensorimotor responses resulting from different rat reactions to visual, acoustic, and tactile stimuli (Ossato et al., 2015; Marti et al., 2019).

##### Evaluation of the Visual Response

Visual response was verified by two behavioral tests, which evaluated the ability of the rat to capture visual information even when the animal is stationary (the visual object response) or when it is moving (the visual placing response). Visual object response test was used to assess the ability of the rat to see an object approaching from the front or side that causes the animal to move or turn the head or withdraw it (Marti et al., 2019). For the frontal visual response, a white horizontal bar was moved frontally to the rat head and the maneuver was repeated three times. For the lateral visual response, a small dentist’s mirror was moved into the rat’s field of view in a horizontal arc, until the stimulus was between the rat’s eyes. The procedure was conducted bilaterally and was repeated three times. The score assigned was a value of 1 if there was a reflection in the rat movement or 0 if not. The total value was calculated by adding the scores obtained in the frontal with that obtained in the lateral visual object response (overall score, 9). Evaluation of the visual object response was measured at 0, 5, 30, and 60 min post-injection. Visual placing response test is performed using a tail suspension modified apparatus able to bring down the rat towards the floor at a constant speed of 10 cm/s (Marti et al., 2019). A camera videotapes the downward movement of the rat. The analysis frame by frame allows evaluating the beginning of the reaction of the rat while it is close to the floor. When the rat starts the reaction, an electronic ruler evaluates the perpendicular distance in millimeters between the eyes of the rat to the floor. The naive rats perceive the floor and it prepares to contact at a distance of about 27 ± 4.5 mm. Evaluation of the visual placing response was measured at 0, 15, 40, and 70 min post-injection.

##### Evaluation of Acoustic Response

Acoustic response measures the reflex of the rat in replay to an acoustic stimulus produced behind the animal. In particular, four acoustic stimuli of different intensities and frequencies were tested (Marti et al., 2019). Each sound test was repeated three times, giving a value of 1 if there was a response and 0 if not present, for a total score of 3 for each sound. The acoustic total score was calculated by adding scores obtained in the four tests (overall score, 12). Evaluation of the visual object response was measured at 0, 10, 30, and 60 min post-injection.

##### Evaluation of Tactile Response

The overall tactile response in the rat was verified through vibrissae, pinna, and corneal reflexes (modified from Ossato et al., 2018; Marti et al., 2019). Vibrissae reflex was evaluated by touching vibrissae (right and left) with a thin hypodermic needle once for side, giving a value of 1 if there was a reflex (turning of the head to the side of touch or vibrissae movement) or 0 if not present (overall score, 2). Evaluation of the vibrissae reflex was measured at 0, 5, 30, and 60 min post-injection. Pinna reflex was assessed by touching pavilions (left and right) with a thin hypodermic needle. First, the interior pavilions and then the external. This test was repeated twice for side, giving a value of 1 if there was a reflex and 0 if not present (overall score, 4). Evaluation of the pinna reflex was measured at 0, 5, 30, and 60 min post-injection. Corneal reflex was assessed gently touching the cornea of the rat with a thin hypodermic needle and evaluating the response, assigning a value of 1 if the rat moved only the head, 2 if it only closed the eyelid, and 3 if it closed the lid and moved the head. The procedure was conducted bilaterally (overall score, 6) and was measured at 0, 5, 30, and 60 min post-injection.

#### Evaluation of Core and Surface Body Temperature

To assess the effects of 25I-NBOMe on thermoregulation, we measured both changes in the core (rectal) and surface (ventral fur) temperature. The core temperature was evaluated by a probe (1-mm diameter) that was gently inserted, after lubrication with liquid vaseline, into the rectum of the rat (to about 2 cm) and left in position until the stabilization of the temperature (about 10 s; Marti et al., 2019). The probe was connected to a Cole Parmer digital thermometer, model 8402. The surface temperature was measured by a Microlife FR 1DZ1 digital infrared thermometer, placed at 1 cm from the surface of the abdomen of the rat (Marti et al., 2019). Core and surface rat body temperatures were measured at 0, 10, 35, and 65 min.

#### Evaluation of Pain Induced by a Mechanical Stimulation of Tail

Acute mechanical nociception was evaluated using the tail and hind paw pinch tests (modified by (Vigolo et al., 2015). A special rigid probe connected to a digital dynamometer (ZP-50N, IMADA, Japan) was gently placed on the tail (in the distal portion) or the hind paw of the rat and a progressive pressure was applied. When the rat flicked its tail or removed the hind paw, the pressure was stopped and the digital instrument saved the maximum peak of weight supported (g/force). A cutoff (500 g/force) was set to avoid tissue damage. The test was repeated three times and the final value was calculated with the average of three obtained scores. Acute mechanical nociception was measured at 0, 15, 40, and 70 min post-injection.

#### Startle and Prepulse Inhibition Analysis

Startle and prepulse inhibition studies were performed as previously reported (Marti et al., 2019). Male and female rats were tested for acoustic startle reactivity in startle chambers (Ugo Basile apparatus, Milan, Italy) consisting of a sound-attenuated, lighted, and ventilated enclosure holding a transparent non-restrictive Perspex^®^ cage (modified version for rats 200 × 90 × 80 mm). A loudspeaker mounted laterally the holder produced all acoustic stimuli. Peak and amplitudes of the startle response were detected by a load cell. At the onset of the startling stimulus, 300-ms readings were recorded and the wave amplitude evoked by the movement of the rat startle response was measured. Acoustic startle test sessions consisted of startle trials (pulse-alone) and prepulse trials (prepulse + pulse). The pulse-alone trial consisted of a 40-ms 120-dB pulse. Prepulse + pulse trials sequence consisted of a 20-ms acoustic prepulse, 80-ms delay, and then a 40-ms 120-dB startle pulse (100-ms onset–onset). There was an average of 15 s (range = 9–21 s) between the trials. Each startle session began with a 10-min acclimation period with a 65-dB broadband white noise that was present continuously throughout the session. The test session contained 40 trials composed by pulse-alone and prepulse + pulse trials (with three different prepulses of 68, 75, and 85 dB) presented in a pseudorandomized order. Male and female rats were placed in the startle chambers 5 min after treatment with 25I-NBOMe. The entire startle/PPI test lasted 20 min. The amount of PPI was expressed as the percentage decrease in the amplitude of the startle reactivity caused by the presentation of the prepulse (% PPI). 25I-NBOMe (0.1–1 mg/kg, i.p.) was administered intraperitoneally and startle/PPI responses were recorded 30 min (including the 10-min acclimation period) after drug injections.

#### Statistical Analysis of Behavioral Tests

Core and surface temperature values are expressed as the difference between control temperature (before injection) and temperature following drug administration (Δ°C). Antinociception (tail pinch tests) is calculated as percent of maximal possible effect {*E*_Max%_ = [(test − control latency)/(cut-off time − control)] × 100}. Data are expressed in absolute values, Δ°C (core and surface temperature), *E*_max%_ (tail pinch tests), and arbitrary units (tail rigidity). In sensorimotor response experiments, data are expressed in arbitrary units (visual objects response, acoustic response, vibrissae, corneal, and pinna reflex) and percentage of baseline (visual placing response). The statistical analyses of the effects of the individual substances in different concentrations over time and that of antagonism studies in histograms were performed by ANOVA (two-way) followed by Bonferroni’s test for multiple comparisons. The statistical analysis was performed with the program Prism software (GraphPad Prism, USA). The amount of PPI was calculated as a percentage score for each prepulse + pulse trial type: %PPI = 100 − {[(startle response for prepulse + pulse trial)/(startle response for pulse-alone trial)] × 100}. Startle magnitude was calculated as the average response to all pulse-alone trials. All the numerical data are given as mean ± SEM. Data were analyzed by utilizing repeated measures ANOVA. The statistical analysis was performed with the program Prism software (GraphPad Prism, USA).

## Results

### Evaluation of Estrous Cycle

Initially, we evaluated the estrous cycle phase prior to the microdialysis experiment, as described in *Materials and Methods*. Three-way ANOVA was performed comparing proestrus–estrus phase with metestrus–diestrus, and no significant differences have been observed. For this reason, we decided to not show those data and combine the female data across the estrous cycle for simplicity.

### Dopamine and Serotonin Basal Levels

**Figure 1:**
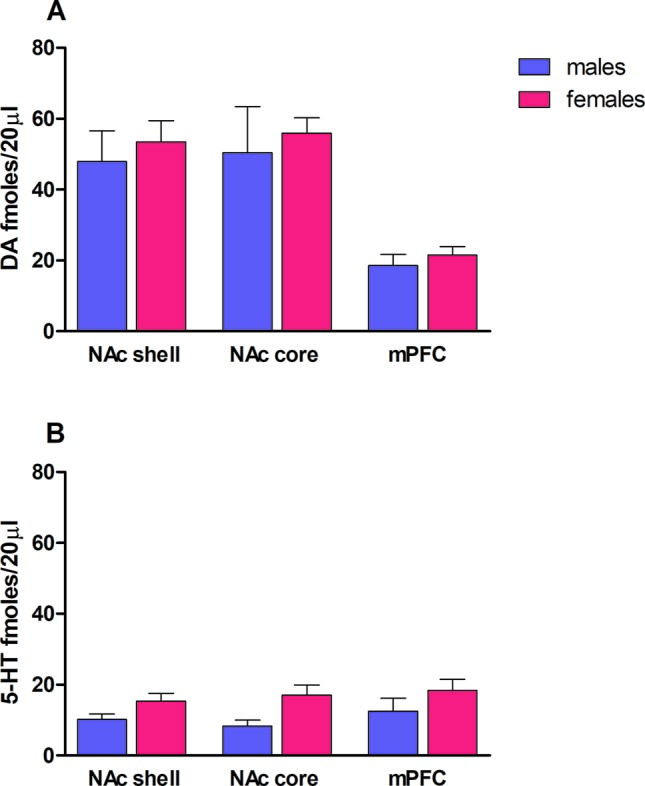
Rat dopamine **(A)** and serotonin **(B)** basal levels in both male (*blue bars*) and female (*magenta bars*) rats. Results are expressed as the mean ± SEM of change in DA/5-HT extracellular levels expressed as fmol/20 μl sample in nucleus accumbens (NAc) shell, NAc core, and mPFC.

Rat basal values of DA, expressed as fmol/20 µl sample (mean ± SEM), were in males: NAc shell 48 ± 9 (*N* = 7), NAc core 50 ± 13 (*N* = 5), mPFC 19 ± 3 (*N* = 8); in females: NAc shell 53 ± 6 (*N* = 23), NAc core 56 ± 4 (*N* = 18), mPFC 22 ± 2 (*N* = 19) (see **Figures 1A**). Rat basal values of 5-HT, expressed as fmol/20 µl sample (mean ± SEM), were in males: NAc shell 10 ± 1 (*N* = 11), NAc core 8 ± 2 (*N* = 8), mPFC 12 ± 4 (*N* = 10); in females: NAc shell 15 ± 2 (*n* = 23), NAc core 17 ± 3 (*n* = 18), mPFC 18 ± 3 (*n* = 21) (see **Figure 1B**). One-way ANOVA was performed for each terminal area, revealing no sex differences in both DA and 5-HT basal outputs (as shown in **Figures 1A, B**).

### Effect of 25I-NBOMe Administration on DA Transmission in the NAc Shell and Core and in the mPFC

#### Males

**Figure 2:**
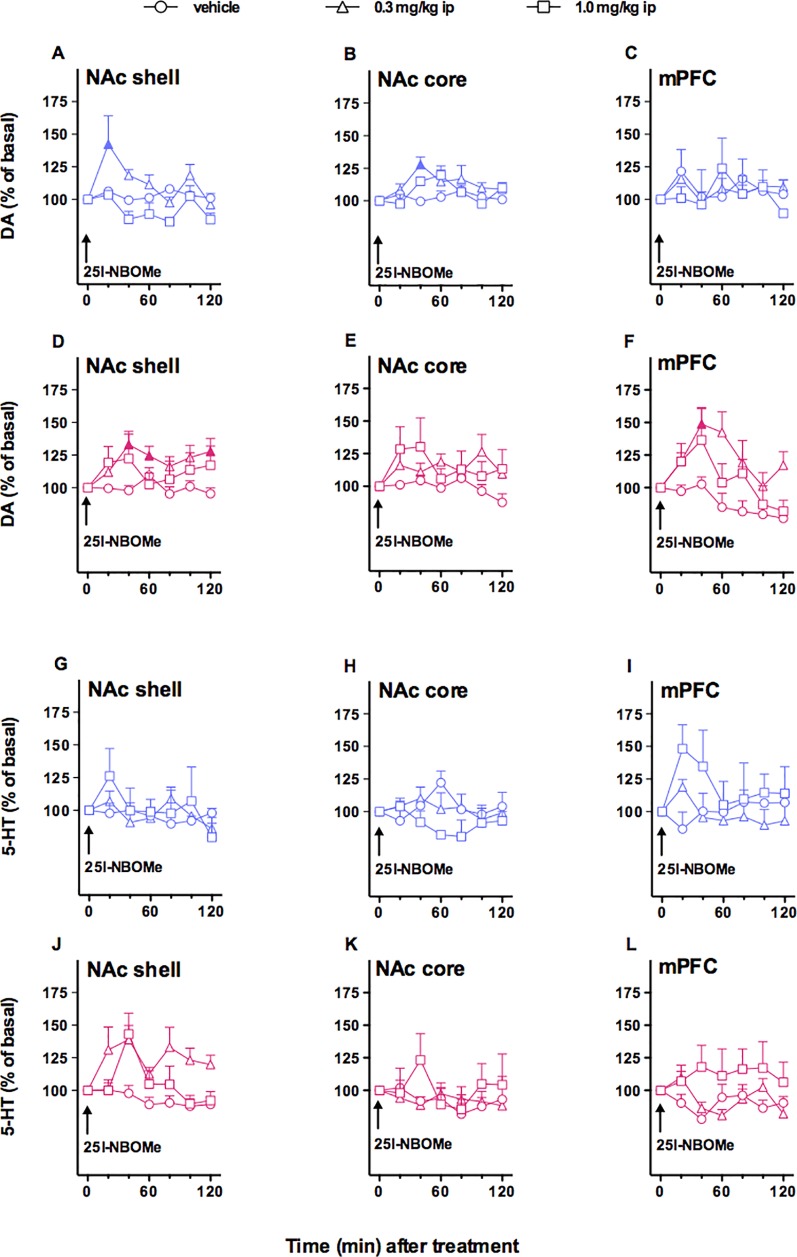
Effect of 4-iodo-2,5-dimethoxy-*N*-(2-methoxybenzyl)phenethylamine (25I-NBOMe) administration (0.3 and 1.0 mg/kg, i.p) on dopamine (DA) **(A**–**F)** and serotonin (5-HT) **(G**–**L)** transmissions in the nucleus accumbens (NAc) shell, NAc core, and medial prefrontal cortex (mPFC) in male (*blue symbols*) and female (*magenta symbols*) rats. Results are expressed as the mean ± SEM of change in DA/5-HT extracellular levels expressed as the percentage of basal values. The *arrow* indicates the start of i.p. injection at of vehicle (*circles*) or 25I-NBOMe 0.3 mg/kg (*triangles*) or 25I-NBOMe 1.0 mg/kg (*squares*) in NAc shell, NAc core, and mPFC. Statistical analysis was performed by three-way or two-way ANOVA followed by the Tukey’s HSD *post hoc* test for multiple comparisons. *Solid symbol*: *p* < 0.05 with respect to basal values (DA males: NAc shell, *N* = 15; NAc core, *N* = 10; mPFC, *N* = 14; DA females: NAc shell, *N* = 34; NAc core, *N* = 31; mPFC, *N* = 27; 5-HT males: NAc shell, *N* = 11; NAc core, *N* = 10; mPFC, *N* = 11; 5-HT females: NAc shell, *N* = 38; NAc core, *N* = 20; mPFC, *N* = 26).

In this experiment, we evaluated the effect of two doses of 25I-NBOMe (0.3 and 1.0 mg/kg, i.p.) on extracellular DA levels in NAc shell and core and the mPFC (**Figures 2A–C**). As shown in **Figure 2**, this phenethylamine affects DA transmission to a small extent only in NAc shell and core, but not in mPFC in male rats. Three-way ANOVA showed a main effect of treatment (*F*_2,30_ = 6.50, *p < 0.05) and time × area interaction (*F*_12,180_ = 2.18, *p < 0.05). In animals implanted in NAc shell, two-way ANOVA showed a main effect of treatment (*F*_2,12_ = 7.79, *p < 0.01) and time (*F*_6,72_ = 3.62,*p < 0.01). Tukey’s *post hoc* tests showed a larger increase of dialysate DA in the NAc shell after 25I-NBOMe 0.3 mg/kg, i.p., revealing differences at the 20-min sample with respect to basal values (**Figure 2A**). In animals implanted in NAc core, two-way ANOVA showed a main effect of time (*F*_6,12_ = 3.10, *p < 0.05). Tukey’s *post hoc* tests showed a larger increase of dialysate DA in the NAc core, revealing differences at the 40-min sample with respect to basal values (**Figure 2B**). In animals implanted in mPFC, two-way ANOVA showed no significant effects (**Figure 2C**).

#### Females

In this experiment, we evaluated the effect of two doses of 25I-NBOMe (0.3 and 1.0 mg/kg, i.p.) on extracellular DA levels in NAc shell and core and the mPFC (**Figures 2D–F**). The dopamine transmission is affected by the administration of the drug in the NAc shell and lightly in the mPFC, but not in the NAc core. Three-way ANOVA showed a main effect of treatment (*F*_2,83_ = 10.33, *p < 0.0001), time (*F*_6,498_ = 6.63, *p < 0.0001), time × area interaction (*F*_12,498_ = 2.55, *p < 0.005), and time × treatment interaction (*F*_12,498_ = 3.08, *p < 0.0005). In animals implanted in NAc shell, two-way ANOVA showed a main effect of treatment (*F*_2,31_ = 3.65, *p < 0.05), and time × treatment interaction (*F*_12,186_ = 2.47, *p < 0.01). Tukey’s *post hoc* tests showed a larger increase of dialysate DA in the NAc 16 shell after 25I-NBOMe 0.3 mg/kg, i.p., revealing differences at the at the 40-, 60-, and 120-min samples with respect to basal values (**Figure 2D**). In animals implanted in NAc core, two-way ANOVA showed no effects (**Figure 2E**). In animals implanted in mPFC, two-way ANOVA showed a main effect of treatment (*F*_1,124_ = 4.17, *p < 0.05) and time (*F*_6,144_ = 5.49, *p < 0.0001). Tukey’s *post hoc* test showed a larger increase of dialysate DA in the mPFC after 25I-NBOMe 0.3 mg/kg, i.p., revealing differences at the 40-min samples with respect to basal values (**Figure 2F**).

In an attempt to compare the effects observed in male and female rats, a four-way ANOVA has been performed. No statistically significant differences between sexes have been shown.

### Effect of 25I-NBOMe Administration on 5-HT Transmission in the NAc Shell and Core and in the mPFC

#### Males

In this experiment, we evaluated the effect of two doses of 25I-NBOMe (0.3 and 1.0 mg/kg, i.p.) on extracellular 5-HT levels in NAc shell and core and in the mPFC. As shown in **Figures 2G–I**, the compound did not affect the serotonergic transmission in all the areas studied. Three-way ANOVA showed a significant time × treatment interaction (*F*_12,156_ = 1.89, *p < 0.05). Two-way ANOVA analysis did not highlight significative differences between vehicle-treated animals and 25I-NBOMe-treated animals either for the three areas.

#### Females

In this experiment, we evaluated the effect of 25I-NBOMe (0.3 and 1.0 mg/kg, i.p.) on extracellular 5-HT levels in NAc shell, NAc core, and mPFC. As shown in **Figures 2J–L**, the compound affects the serotonergic transmission to a small extent only in NAc shell. Three-way ANOVA showed a main effect of area (*F*_2,75_ = 14.28, *p < 0.05), treatment (*F*_2,75_ = 4.58, *p < 0.05), and area × treatment interaction (*F*_42,75_ = 4.9, *p < 0.005). Tukey’s *post hoc* tests showed no differences. In animals implanted in NAc shell (**Figure 2J**), two-way ANOVA showed a main effect of treatment (*F*_2,35_ = 15.24, *p < 0.0001) and time (*F*_6,210_ = 3.12, *p < 0.01), but no significant differences were revealed by Tukey’s *post hoc* test. Two-way ANOVA analysis did not highlight significant differences between vehicle-treated animals and 25I-NBOMe-treated animals for the NAc core (**Figure 2K**). In animals implanted in the mPFC (**Figure 2L**), two-way ANOVA showed a main effect of treatment (*F*_2,23_ = 3.48, *p < 0,05), without any significant results in the Tukey’s *post hoc* test. In an attempt to compare the effects observed in male and female rats, a four-way ANOVA has been performed. No statistically significant differences between sexes have been shown.

### Effects of 25I-NBOMe on Behavioral Tests

#### Sensorimotor Studies

##### Evaluation of the Visual Object Response

**Figure 3:**
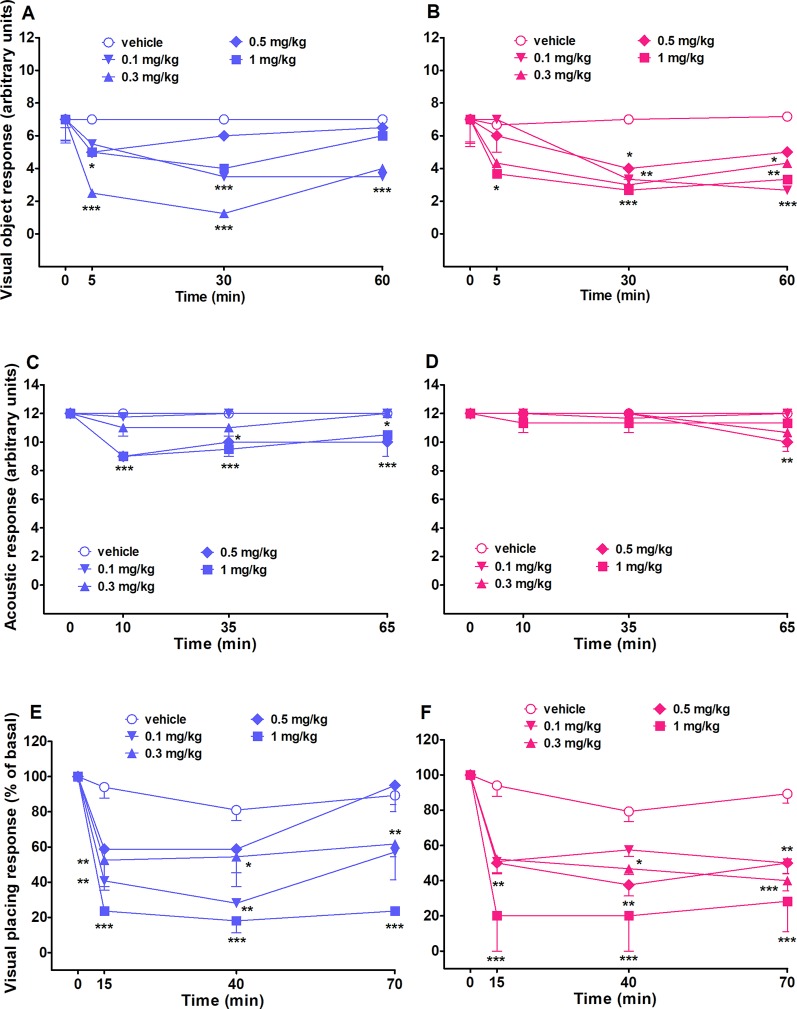
Intraperitoneal injection (0.1–1 mg/kg) of 25I-NBOMe in male and female rats on the visual object test **(A** and **B)**, on the acoustic response **(C** and **D)**, and on the visual placing test **(E** and **F)**. Data are expressed as arbitrary units **(A**, **B**, **C**, and **D)** or percentage of basal **(E** and **F)** and represent the mean ± SEM of six determinations for each treatment. Statistical analysis was performed by two-way ANOVA followed by Bonferroni’s test for multiple comparisons for the dose–response curve at different times. *p < 0.05, **p < 0.01, ***p < 0.001 versus vehicle.

Visual object response did not change in both vehicle-treated male and female rats over 60 minutes Of observation (**Figures 3A, B**). Systemic administration of 25I-NBOMe (0.1–1 mg/kg, i.p.) reduced the visual object response in both sex rats and the effect persisted up to 60 min (**Figures 3A, B**). Two-way ANOVA followed by the Bonferroni’s test for multiple comparisons in male rats showed a significant effect of treatment (*F*_4,140_ = 22.24, *p* < 0.0001), time (*F*_3,140_ = 22.47, *p* < 0.0001), and time × treatment interaction (*F*_12,140_ = 4.478, *p* < 0.0001). The same statistical analysis for female rats showed a significant effect of treatment (*F*_4,140_ = 8.207, *p* < 0.0001), time (*F*_3,140_ = 15.79, *p* < 0.0001), and time × treatment interaction (*F*_12,140_ = 2.149, *p* < 0.05).

##### Evaluation of the Acoustic Response

Acoustic response did not change in both vehicle-treated male and female rats over 60 min of observation (**Figures 3C, D**). Systemic administration of 25I-NBOMe impairs the acoustic response only in male rats at the two highest doses tested, 0.5 and 1 mg/kg, and this effect is persistent up to 60 min after the treatment (**Figure 3C**). Two-way ANOVA for male rats showed a significant effect of treatment (*F*_4,140_ = 14.54, *p* < 0.0001), time (*F*_3,140_ = 9.144, *p* < 0.0001), and time × treatment interaction (*F*_12,140_ = 2.061, *p* < 0.05). The acoustic response was not inhibited in female rats by 25I-NBOMe. Two-way analysis showed a significant effect of time (*F*_3,140_ = 3.694, *p* < 0.05), and Bonferroni’s test for multiple comparisons showed a tardive little effect displayed by the dose of 0.5 mg/kg, i.p., at 60 min (**Figure 3C, D**).

##### Evaluation of the Visual Placing Response

Visual placing response slightly decreased in both vehicle-treated male and female rats over 70 min of observation (∼17% of reduction at 70 min; **Figures 3E, F**). Systemic administration of 25I-NBOMe reduced the visual placing response in both rat sexes at all the doses tested (0.1–1 mg/kg, i.p.) and the effect persisted up to 70 min, as shown in **Figure 3E**. Two-way analysis showed a significant effect of treatment (*F*_4,140_ = 17.25, *p* < 0.0001), time (*F*_3,140_ = 31.63, *p* < 0.0001), and time × treatment interaction (*F*_12,140_ = 2.582, *p* < 0.005) for male rats. For female rat, as shown in **Figure 3F** statistical analysis showed a significant effect of treatment (*F*_4,140_ = 16.23, *p* < 0.0001), time (*F*_3,140_ = 39.89, *p* < 0.0001), and time × treatment interaction (*F*_12,140_ = 2.135, *p* < 0.05).

##### Evaluation of the Tactile Response

**Figure 4:**
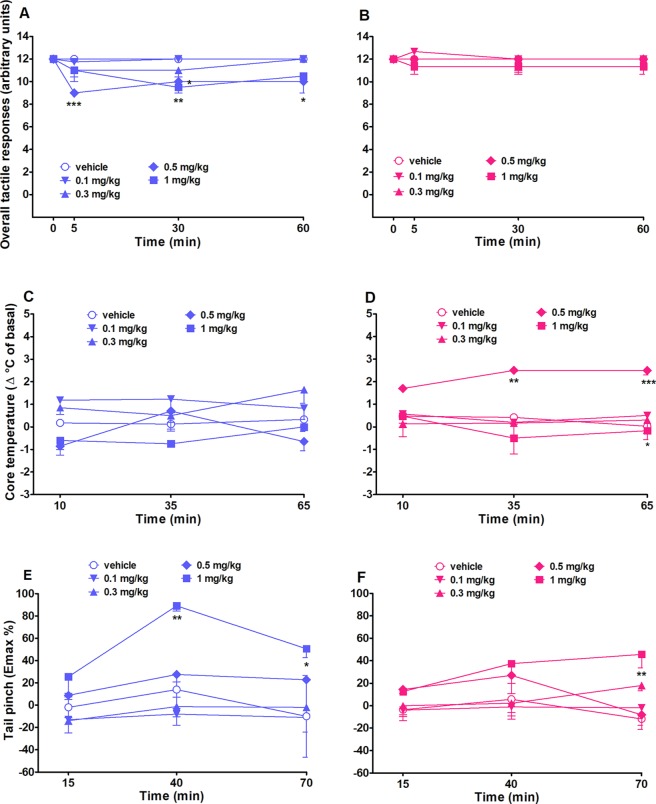
Intraperitoneal injection (0.1–1 mg/kg) of 25I-NBOMe in male and female rats on the overall tactile responses **(A** and **B)** and on core temperature **(C** and **D)** and tail pinch test **(E** and **F)**. Data are expressed as arbitrary units **(A** and **B)**, as difference between control temperature (before injection) and temperature following drug administration (Δ°C; see *Material and Methods*) **(B** and **C)**, or as percentage of maximum effect (*E*_max%_; (see *Material and Methods*) **(E** and **F)** and represent the mean ± SEM of six animals for each treatment. Statistical analysis was performed by two-way ANOVA followed by Bonferroni’s test for multiple comparisons for the dose–response curve of each compound at different times. *p < 0.05, **p < 0.01, ***p < 0.001 versus vehicle.

Overall tactile responses did not change in both vehicle-treated male and female rats over 65 min of observation (**Figures 4A, B**). As shown in **Figure 4A**, intraperitoneal injection (0.1–1 mg/kg) of 25I-NBOMe affected male tactile responses with a significant effect of treatment (*F*_4,140_ = 8.942, *p* < 0.0001) and time (*F*_3,140_ = 4.916, *p* < 0.05). No effects on females’ tactile responses were observed (**Figure 4B**).

#### Evaluation of Core and Surface Body Temperature

Core body temperature did not change in both vehicle-treated male and female rats over 65 min of observation (**Figures 4C, D**). Systemic administration of 25I-NBOMe (0.1–1 mg/kg, i.p.) did not affect core (**Figure 4C**) body temperatures in male rats. Two-way ANOVA showed a significant effect of treatment (*F*_4,105_ = 8.880, *p* < 0.0001). The dose of 0.5 mg/kg, i.p., affected significantly the core temperature in female rats (**Figure 4D**), with a significant effect of treatment (*F*_4,105_ = 12.07, *p* < 0.0001). 25I-NBOMe did not affect the surface temperature in male rats; neither in females (data not shown).

#### Evaluation of Pain Induced by a Mechanical Stimulus

The threshold to acute mechanical pain stimulus did not change in both vehicle-treated male and female rats over 70 min of observation (**Figures 4E, F**). Systemic administration of the highest dose of 25I-NBOMe (1 mg/kg, i.p.) heavily increased the threshold to acute mechanical pain stimulus in male rats in the tail pinch test [significant effect of treatment (*F*_4,105_ = 9.822, *p* < 0.001) and time (*F*_2,105_ = 3.110, *p* < 0.05] (**Figure 4E**), whereas in female rats there is a lower effect with the same dose (**Figure 4F**). Statistical analysis showed a significant effect of treatment (*F*_4,105_ = 4.988, *p* < 0.001).

#### Startle/Prepulse Inhibition Studies

**Figure 5:**
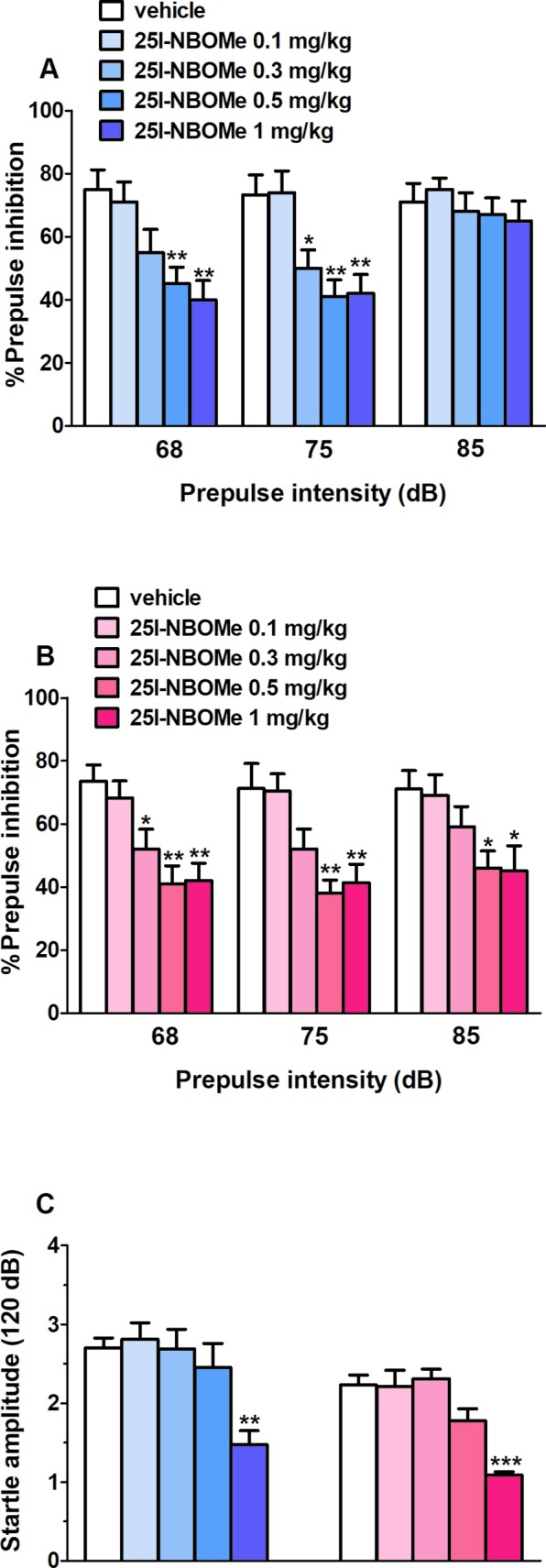
Effect of the systemic administration of 25I-NBOMe (0.1–1 mg/kg, i.p.) on prepulse inhibition (PPI) in male **(A)** and female **(B)** rats and on startle amplitude for both sexes **(C)**. Effects on PPI are shown for the three prepulse intensities (68, 75, and 85 dB) 30 min after treatment **(A** and **B)**. Data are expressed (see *Material and Methods*) as percentage decrease in the amplitude of the startle reactivity caused by presentation of the prepulse (% PPI) **(A** and **B)** and absolute values (in dB) **(C)**. Values represent the mean ± SEM of seven animals for each treatment. Statistical analysis was performed by one-way ANOVA followed by Bonferroni’s test for multiple comparisons. *p < 0.05, **p < 0.01, ***p < 0.001 versus vehicle.

Vehicle injection did not change startle/PPI response in male and female rats, and the effect was similar in naive untreated animals (data not shown). Administration of 25I-NBOMe (0.1–1 mg/kg, i.p.) inhibited the PPI in male rats at 68 dB (*F*_4,30_ = 6.072, *p* < 0.001) and 75 dB (*F*_4,30_ = 7.266, *p* < 0.001) of prepulse intensity (**Figure 5A**), while it inhibited the PPI in female rats at 68 dB (*F*_4,30_ = 7.046, *p* < 0.001), 75 dB (*F*_4,30_ = 6.635, *p* < 0.001), and 85 dB (*F*_4,30_ = 3.501, *p* < 0.05) of prepulse intensity (**Figure 5B**). Moreover, 25I-NBOMe impaired the startle amplitude in male (about ∼50% inhibition; *F*_4,30_ = 5.98, *p* < 0.05) and female (about ∼50% inhibition; *F*_4,30_ = 13.07, *p* < 0.0001) rats at 1 mg/kg at 30 min after drug administration (**Figure 5C**).

## Discussion

The psychedelic compound 25I-NBOMe belongs to the phenethylamines that are a class of NPS spread among youth, with greater diffusion in girls than in boys (Wu et al., 2010; UNODC, 2016). 25I-NBOMe is a 5-HT2A receptor agonist used as a legal substitute of LSD and to mimic the effect of methamphetamine as well (Le Roux et al., 2015; Palamar et al., 2016). In this preclinical study, we evaluated the dopamine (DA) and serotonin (5-HT) releasing properties and the behavioral effects of 25I-NBOMe. Our results showed that 25I-NBOMe affects the DA transmission in the shell of the NAc in both sexes, while the higher variability on the serotonergic transmission’s response, compared to DArgic response, leads to a lack of significative effect when analyzed by three- or two-way ANOVA. However, behavioral data showed that this compound causes visual alterations in both sexes, whereas core temperature is heavily affected in females, and the highest dose tested exerts an analgesic effect particularly prominent in male rats. Moreover, it impairs the startle amplitude and inhibits the PPI in both sexes.

### 25I-NBOMe Affects the Dopaminergic Transmission in the NAc Shell of Both Sexes

It is well established that variability of the basal output may affect the extent of increasing or decreasing in neurotransmitters in brain areas (Di Chiara (1990); De Luca et al., 2018). Therefore, we started comparing DA and 5-HT basal outputs in male and female rats, and we did not observe any differences in any of the brain areas studied (i.e., NAc shell and core and the mPFC), in agreement with previous microdialysis studies aimed at CPu of male and female groups (Xiao and Becker, 1994). Oppositely, Lazenka et al. (2017) observed a lower baseline NAc DA levels in female compared to male rats. Results obtained by monitoring estrous cycle in female rats suggested that, although the treatment affected significantly the DA transmission in all the areas studied and the 5-HT transmission in the NAc shell, estrous cycle phases are not effective in changing the effects of 25I-NBOMe, at least in the present set of experiments. Therefore, we combined the female data across the estrous cycle. Our *in vivo* microdialysis studies showed that the lowest dose of 25I-NBOMe tested (0.3 mg/kg, i.p.) affects the DA transmission in male rats in the NAc shell, with a maximal peak of 36% over basal value, 20 min after the injection, in the NAc core, with an extent of 27% at the 40-min sample, whereas it has no effect on the mPFC DA transmission (both doses). No effect has been observed in the 5-HT transmission in all the three areas tested, with both doses in male rats. The dose of 0.3 mg/kg, i.p., was active in female rats as well, increasing both DA and 5-HT dialysates in the NAc shell, with a maximal peak of 30% over basal value 40 min after the administration, and this effect lasted more than 2 h after the drug administration, whereas in the mPFC only DA extracellular levels are increased with an extent of 45%, and the highest dose of 1.0 mg/kg, i.p., did not show any effect in female rats. It is well known that all the drugs of abuse exert their rewarding effect increasing the DA transmission preferentially in the shell of NAc (Di Chiara et al., 2004; De Luca et al., 2015). Therefore, these results suggest that this compound has a common and alarming feature with them. These results are in line with previous studies showing an increased *ex vivo* striatal DA levels (Jeon et al., 2019) as well as an increased DA transmission in cortical areas of male rats (Herian et al., 2019). Moreover, 25I-NBOMe seems to act differently on DA and 5-HT levels in male and female rats, highlighting sex differences that might influence the frequency of ingestion, as well as the psychoactive effects and the long-term effects. Notably, the higher dose tested was not effective in increasing the DA level; this effect can be due to the formation of active metabolites acting as 5-HT2A antagonists, or it can be the result of off-target effects on other receptors, as previously suggested for other NPS classes (e.g., synthetic cannabinoids) (De Luca et al., 2016). Sex differences have been reported in the initiation of drug use, affecting the continuation of drug use as well as the phases of abstinence and relapse (Becker and Hu, 2008; Fattore et al., 2010), but also in the codification of reinforcement and related cues (Zlebnik, 2019). The greater increase of DA extracellular levels in females compared to males is consistent with previous studies reporting that amphetamine (Virdee et al., 2014), cocaine (Holly et al., 2012), and MDMA (Lazenka et al., 2017) are more effective in increasing DA release in the NAc of female rats. The reason for these neurochemical sex discrepancies has been historically ascribed to deep biological differences, such as sex dimorphisms in the anatomy of DArgic systems in areas like SN and VTA (Walker et al., 2012), as well as ovarian hormone fluctuations (Becker and Hu, 2008). Furthermore, other factors, as pharmacokinetic (Fonsart et al., 2009), pharmacodynamic, and sociocultural differences, have been proposed to take part in the propensity to addiction (Franconi et al., 2013). Additionally, it has been widely demonstrated that female rats exhibit greater sensitivity to psychostimulants compared to males (Walker et al., 2012), with several experimental paradigms such as self-administration and conditioned place preference (Savageau and Beatty, 1981; Becker et al., 1982; Lynch and Carroll, 1999; Walker et al., 2000; Russo et al., 2003; Roth and Carroll, 2004; Harrod et al., 2005; Kantak et al., 2007). Even if we did not observe differences in each stage of the estrous cycle, further investigations are necessary to examine in depth the role of hormones in mediating the effects of this compound. These differences among males and females in responding to these synthetic compounds could explain recent surveys reporting that adolescent girls are more likely, compared to boys, to be ecstasy and/or other hallucinogen users (Wu et al., 2010). In addition, it has also been reported that a given dose of MDMA tends to produce more intense negative psychoactive effects in women than in men (Liechti et al., 2001) and that girls may generally be more vulnerable than boys to developing symptoms of hallucinogen dependence (Wu et al., 2009).

### 25I-NBOMe Causes Visual Alterations in Both Sexes

Data obtained showed that this compound decreases visual responses, causing dangerous visual alterations in both sexes. Sensorimotor alterations, especially visual ones, may be due to the pro-hallucinogenic action of 25I-NBOMe (Halberstadt and Geyer, 2014) and as typically reported for other 5-HT2A agonists (Canal and Morgan, 2012; Halberstadt and Geyer, 2013). All such hallucinogenic compounds exhibit high affinity for 5-HT2A receptors (Roth et al., 1998; González-Maeso and Sealfon, 2009). In fact, genetic or pharmacological inactivation of 5-HT2A receptor signaling blocks the behavioral effects of hallucinogenic compounds in a variety of species, including mice, rats, and humans (Fiorella et al., 1995; Vollenweider et al., 1998; González-Maeso et al., 2003; González-Maeso et al., 2007). Taken together, these findings indicate that 25I-NBOMe, by activating 5-HT2A receptor in cortico-visual circuits, could impair sensorimotor responses by promoting a hallucinogenic state.

### 25I-NBOMe Effects on the Acoustic and Tactile Responses

The decrease in the acoustic response is consistent with previous studies demonstrating that the administration of the analogue (±)-2,5-Dimethoxy-4- iodoamphetamine (DOI), in the dose range of 0.25–1.0 mg/kg, disrupted the startle response in Sprague–Dawley rats (Swerdlow et al., 2006). Recently, it has been demonstrated the role of 5-HT in modulating auditory brainstem responses in mice, starting from the cochlear nucleus (Papesh and Hurley, 2016); indeed, in the dorsal region of this nucleus, the activation of 5-HT2 receptors acts increasing the electrical activity of neurons, leading to a final suppression of auditory process (Felix et al., 2017; Tang and Trussell, 2017). Moreover, it has been recently shown that MDMA reduces acoustic and tactile responses as well (Marti et al., 2019), and this is a 5-HT2 receptor-mediated effect (Geyer and Tapson, 1988).

### 25I-NBOMe Effects on Body Temperature

Hyperthermia, which is one of the symptoms of the serotonin syndrome, was observed only in females with the dose of 0.5 mg/kg. This difference can be related to a distinct pharmacokinetic and pharmacodynamic compared to males, as previously described for other substances, which included amphetamine (Brady and Randall, 1999; Becker et al., 2001; Lynch et al., 2002; Carroll et al., 2004). Importantly, these results could explain why MDMA and hallucinogens seem to be more effective in women compared to men (Liechti et al., 2001; Wu et al., 2010).

### 25I-NBOMe Has a Greater Analgesic in Males

The highest dose tested (1 mg/kg, i.p.) exerts an analgesic effect prominent in male rats and minor in female rats, increasing the threshold to acute mechanical pain stimulus. This effect in male rats is higher than the effect obtained with compounds acting by the cannabinoid pathway (De Luca et al., 2015; Vigolo et al., 2015). This compound has a great affinity for rat 5-HT2A receptors (Ki = 0.087 nM) (Braden et al., 2006), but it has lower affinity also for µ-opioid receptors (Ki = 82 nM) and Ki greater than 500 nM for 5-HT1A receptors (Nichols et al., 2008). Therefore, it is possible to assume that the highest dose tested binds 5-HT2A receptors first, and further with other receptors such as 5-HT1A and µ-receptors, producing the analgesic effect. It is well known that serotonergic pathways running from the brainstem to the spinal cord are considered to be essential to the mechanisms of descending pain controls (Mayer et al., 1971; Zemlan et al., 1980; Clatworthy et al., 1988; Fields et al., 1991), and 5-HT2A receptors appear to play a critical role on nociceptive responses (Bardin et al., 2000; Sasaki et al., 2001; Kjørsvik et al., 2001). In particular, the administration of 5-HT2A receptor agonist DOI mediates antinociceptive effects in the craniofacial nociception (Okamoto et al., 2007). Different nociceptive responses have been observed before (Gamaro et al., 2014), and they are probably due to sex dimorphism in the localization of serotonergic receptors (Araldi et al., 2017), as well as different microglia activation within the periaqueductal gray (Doyle et al., 2017). Indeed, the activation of 5-HT2A receptors has been demonstrated to stimulate the secretion of various hormones (Van de Kar et al., 2001), among these the estradiol in both animals and humans (Moses et al., 2000; Kugaya et al., 2003; Frokjaer et al., 2010; Moses-Kolko et al., 2011). It is well known that estradiol is able to increase mechanical pain threshold in both sexes (Lu et al., 2012) while estriol fails it. The reason for this could be the ability of females to metabolize estradiol in estriol more quickly than males.

### 25I-NBOMe Impairs the Acoustic Startle Reflex in Male and Female Rats

Notably, 25I-NBOMe in our experiments showed to impair the acoustic startle reflex with the same extent (about 50% inhibition) in both sexes at the highest dose of 1 mg/kg, i.p., and to disrupt the sensorimotor gating significantly in both male and females compared to the vehicle-treated group (dose, 0.3–1 mg/kg, i.p.), with a tendency to an increased impairment at the prepulse intensity of 85 dB in females compared to males. It has been shown before that LSD exerts the same effects on PPI by activating 5-HT2A receptors in rats (Halberstadt and Geyer, 2010), and MDMA as well in both rats and mice (Marti et al., 2019). These results are alarming since the PPI has been widely defined as a marker of vulnerability to develop a neuropsychiatric disorder (Marti et al., 2019).

## Conclusions

In conclusion, we have shown that the synthetic hallucinogen 25I-NBOMe affects the DArgic transmission in the NAc shell in a sex-independent manner, while mPFC DA seems to be more responsive in females compared to males. However, behavioral data proved that the severity of side effects occurring after 25I-NBOMe ingestion, probably mediated by serotonin pathways, can be different in male and female rats, as suggested also by a tendency, although not statistically significant, to a preferential increase of extracellular 5-HT in the NAc shell and a long-lasting stimulation in the mPFC of females compared to males. Regrettably, the experimental conditions of the present study have not been adequate to correlate either basal or 25I-NBOMe-stimulated brain levels of DA and 5-HT with hormonal fluctuations. In order to expand our knowledge on sex differences in the response to NPS, further experiments, most likely based on the direct evaluation of blood levels of hormones instead of indirect estimation by vaginal smears, would be needed. On the other hand, the observation of a higher core temperature in female rats and a marked analgesic effect in male rats after the administration of 25I-NBOMe may account for a gender-specific toxicity, thus highlighting possible distinct pharmacokinetics and pharmacodynamics as well as impact of enzyme genotype among sexes. These features suggest that the habit of consuming the psychedelic agent 25I-NBOMe and its analogues (Gee et al., 2016) pose a high risk of developing hyperthermia (i.e., serotonin syndrome) and has altered nociceptive responses. Moreover, the decreased acoustic reflex and the impaired visual responses observed in this preclinical study, coupled with the unawareness of what is going to be ingested in humans, pose a significant issue for public health and safety. Notably, in 2016, over 10 million people have been reported to drive under the influence of illicit drugs (DUID) (Substance Abuse and Mental Health Services Administration, 2017), and the impairment of visual and acoustic reflexes may clearly lead to fatal DUID, as reported after the ingestion of NBOMes (Rajotte et al., 2017). Although the findings of the present research give us important preclinical information, further investigations are necessary to clarify sex differences in toxicological responses to different drugs. Moreover, studies including pharmacological, toxicological, and forensic evidence at both preclinical and clinical levels are needed in order to more widely profile NPS effects and intoxication.

## Data Availability Statement

The datasets generated for this study are available on request to the corresponding author.

## Ethics Statement

All animal experiments were carried out in accordance with the Guidelines for the Care and Use of Mammals in Neuroscience and Behavioral Research according to Italian (D.L. 116/92 and 152/06) and European Council directives (609/86 and 63/2010) and in compliance with the approved animal policies by the Ethical Committee for Animal Experiments (CESA, University of Cagliari) and the Italian Ministry of Health (Aut. N. 162/2016- PR; Aut. N.352/2015-PR).

## Author Contributions

CM designed the experiment, and wrote the first draft of the paper. CM and NP performed the microdialysis experiments and the data analysis. MM, MT and RA contributed with all the behavioral experiments and figures. MM aided in interpreting the results and worked on the manuscript. MPC provided useful contribution to the content and substantially revised the manuscript. MDL conceived the topic, supervised and coordinated the work and wrote the final version of the manuscript. All the coauthors contributed to the present piece of work before approving it for final submission.

## Conflict of Interest

The authors declare that the research was conducted in the absence of any commercial or financial relationships that could be construed as a potential conflict of interest.

The reviewer LO declared a past co-authorship with one of the authors MDL to the handling editor.

## Abbreviations

DA, Dopamine/dopaminergic; DOI, (±)-2,5-Dimethoxy-4- iodoamphetamine; HPLC, High-performance liquid chromatography; 25I-NBOMe, 4-Iodo-2,5-dimethoxy-N-(2-methoxybenzyl)phenethylamine; LSD, Lysergic acid diethylamide; MDMA, 3,4-Methylenedioxy-methamphetamine; mPFC, Medial prefrontal cortex; NAc, Nucleus accumbens; NPS, Novel psychoactive substances; PPI, Prepulse inhibition; 5-HT, Serotonin/serotonergic.
